# Retraction Notice to: Upregulation of OIP5-AS1 Predicts Poor Prognosis and Contributes to Thyroid Cancer Cell Proliferation and Migration

**DOI:** 10.1016/j.omtn.2022.01.023

**Published:** 2022-02-03

**Authors:** Qiuli Li, Weichao Chen, Rongzhen Luo, Zhiyi Zhang, Ming Song, Wenkuan Chen, Zhongyuan Yang, Yuanzhong Yang, Zhuming Guo, Ankui Yang

## Main text

(Molecular Therapy: Nucleic Acids *20*, 279–291; June 2020)

The corresponding author requested extensive changes to the manuscript following its publication, including the removal of all patient data from the manuscript, encompassing the removal of Figures 1A, 1C, 4E, 5H, and S3F and Tables 1 and 2, as well as substitutions of Figures 7E and 8. After reviewing these requests, the Editors feel the findings of the manuscript can no longer be relied upon and have decided to retract the paper. The corresponding author has agreed to the retraction.

